# The Immune Escape Strategy of Rabies Virus and Its Pathogenicity Mechanisms

**DOI:** 10.3390/v16111774

**Published:** 2024-11-14

**Authors:** Abraha Bahlbi Kiflu

**Affiliations:** 1 State Key Laboratory for Conservation and Utilization of Subtropical Agro-Bioresources, Guangxi University, Nanning 530004, China; abrahabahlbikiflu@st.gxu.edu.cn; 2 College of Animal Sciences and Veterinary Medicine, Guangxi University, Nanning 530004, China

**Keywords:** RABV, immune evasion, IFN-α/β, cytkines/chemokines, BBB permeability, apoptosis, autophagy, mitochondrial dysfunction, INOS, neurons

## Abstract

In contrast to most other rhabdoviruses, which spread by insect vectors, the rabies virus (RABV) is a very unusual member of the Rhabdoviridae family, since it has evolved to be fully adapted to warm-blooded hosts and spread directly between them. There are differences in the immune responses to laboratory-attenuated RABV and wild-type rabies virus infections. Various investigations showed that whilst laboratory-attenuated RABV elicits an innate immune response, wild-type RABV evades detection. Pathogenic RABV infection bypasses immune response by antagonizing interferon induction, which prevents downstream signal activation and impairs antiviral proteins and inflammatory cytokines production that could eliminate the virus. On the contrary, non-pathogenic RABV infection leads to immune activation and suppresses the disease. Apart from that, through recruiting leukocytes into the central nervous system (CNS) and enhancing the blood–brain barrier (BBB) permeability, which are vital factors for viral clearance and protection, cytokines/chemokines released during RABV infection play a critical role in suppressing the disease. Furthermore, early apoptosis of neural cells limit replication and spread of avirulent RABV infection, but street RABV strains infection cause delayed apoptosis that help them spread further to healthy cells and circumvent early immune exposure. Similarly, a cellular regulation mechanism called autophagy eliminates unused or damaged cytoplasmic materials and destroy microbes by delivering them to the lysosomes as part of a nonspecific immune defense mechanism. Infection with laboratory fixed RABV strains lead to complete autophagy and the viruses are eliminated. But incomplete autophagy during pathogenic RABV infection failed to destroy the viruses and might aid the virus in dodging detection by antigen-presenting cells, which could otherwise elicit adaptive immune activation. Pathogenic RABV P and M proteins, as well as high concentration of nitric oxide, which is produced during rabies virus infection, inhibits activities of mitochondrial proteins, which triggers the generation of reactive oxygen species, resulting in oxidative stress, contributing to mitochondrial malfunction and, finally, neuron process degeneration.

## 1. Introduction

**Figure 1:**
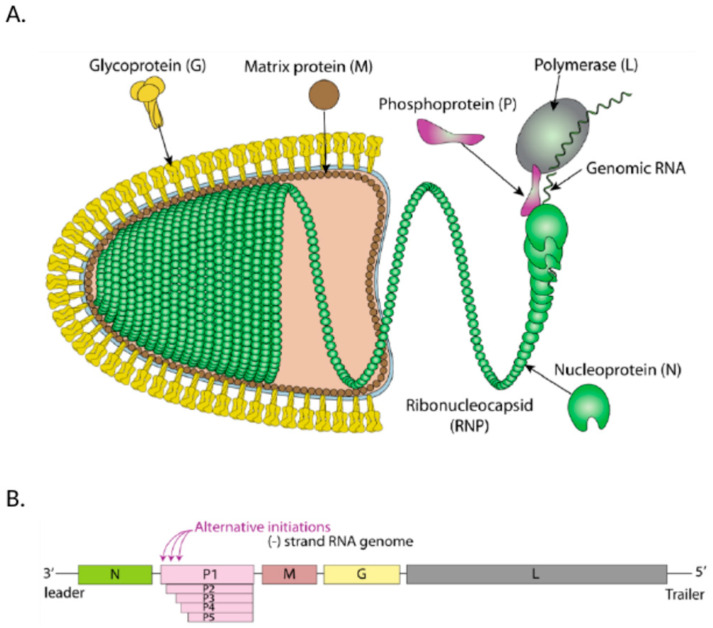
(**A**). RABV virion. Rabies virus is enveloped, bullet-shaped, with a body size of about 180 × 75 nm. (**B**). RABV genome. The RNA genome of the RABV is single-stranded and non-segmented, with a genome size of about 12 kb. It contains a leader and trailer region at the 3′ and 5′ ends, along with five structural proteins (N, P, M, G, and L) and four intergenic non-coding sequences. Multiple P proteins (P (P1), P2, P3, P4 and P5) are produced through alternative initiation from in-frame AUG start codons due to a leaky scanning mechanism. (Original source of the image; Philippe Le Mercier, SIB Swiss Institute of Bioinformatics) [9].

Rabies is a highly lethal zoonotic illness with a mortality rate of approaching 100% and is caused by the rabies virus. Despite global efforts in prevention and control, a small percentage of cases persist in wealthy nations [1]. The disease remains prevalent in numerous low-income countries, mainly in Asia, South America, and Africa [2]. Rabies kills over 59,000 people annually, with no effective medications available, except for vaccination and immediate treatment with specialized antiserum [3]. The inadequate understanding of RABV’s pathogenic mechanism and its relationship with the host defense reaction hinders the development of therapeutic medications. RABV is a non-segmented, single-stranded, negative-sense RNA virus capable of infecting all warm-blooded animals due to its wide host range [4]. The genomic and anti-genomic RNA are encapsulated by N proteins, and the resulting ribonucleoprotein (RNP) shields RNA from RNases destruction [5]. RABV P protein, a co-factor of RNA polymerase, forms a link between the ribonucleoprotein (RNP) and polymerase (L protein) in the polymerase complex [5]. Research shows that P protein’s mRNA synthesizes four N-terminally truncated P products (P2, P3, P4, as well as P5) through a leaky scanning process at internal methionine nucleotides [6] Figure 1.The RABV M protein binds to the RABV G protein and ribonucleoprotein (RNP), facilitating viral budding by sequestering it in a cell membrane, forming the sole protein on the rabies virus (RABV) virion’s outer surface [5]. Viral entrances into the cell via cell receptor-mediated endocytosis depends on the G protein’s outer membrane region recognizing and adhering to the host cell receptors [7]. The neuronal cell adhesion molecule (NCAM), the p75 neurotrophic receptor (p75NTR), and the nicotinic acetylcholine receptor (nAChR) facilitate RABV entry into cells [8]. The G protein is the main target of virus-neutralizing antibodies (VNA), protecting against rabies disease. It interacts with host cell receptors and facilitates pH-triggered fusion, releasing RNP into host cell’s cytoplasm [5]. Mammalian cells cannot transcribe negative-stranded RNA, so the rabies virus (RABV) produces its own RNA-dependent RNA polymerase, or L protein, to carry out transcription. The ribosomes cannot read negative sense RNA, so the negative sense strand must first be transcribed into a complementary positive sense strand before protein synthesis can occur. The rabies virus (RABV) genome and anti-genome is required to be encased in the nucleoprotein for replication by the viral RNA-dependent RNA polymerase (L) and its cofactor. Once enough viral proteins are produced, the polymerase switches to replication mode, creating new copies of positive-sense RNA that serves as templates for more viral RNA. These RNA-protein complexes then assemble into new virions at the cell membrane [5]. Rabies treatment is challenging due to its neurotropic nature, immune privileged sites, and ability to spread to many host species. The virus suppresses immune responses and causes neuron degeneration, leading to paralysis and death. Despite its long history, effective therapy remains elusive.

## 2. Antagonism of Interferon Production by RABV Proteins

Interferons are cytokines with antiviral properties which play a critical role in defending against viral infections and monitoring for cancer cells. There are two main groups, type I and type II IFNs [10]. There are many type I interferons (IFNs) with similar structures, such as IFN-α (13 sub-types), IFN-β, IFN-κ, IFN-τ, IFN-δ, IFN-ε, and IFN-ω [11]. The retinoic acid inducible gene 1(RIG-I) is a cytoplasm RNA sensor that identifies 5′ viral triphosphate single-stranded RNA during infection, activating IKKε and TBK-1. This leads to the activation of IRF3 and IRF7 that relocate to the nucleus to induce the production of IFN α/β. IFNγ can also be generated by cytokines or the activation of other receptors when there is infection or tissue damage [12]. IFN-α/β plus IFN-γ bind to IFN-α receptor and IFN-γ receptors, activating the JAK-STAT signaling pathway, phosphorylating STAT1-3 and dimerize it [13]. Interferon-stimulated gene factor 3 (ISGF3) is a transcriptional complex consisting of STAT1, STAT2 and IRF-9 that translocate to the nucleus in response to interferon stimulation [14]. This complex binds to DNA sequences like gamma-activated sequences (GAS) and interferon-stimulated response element (ISRE), promoting the release of interferon stimulated genes (ISGs) with anti-inflammatory and antiviral properties [15]. RABV has evolved different mechanisms to bypass the immune response, such as inhibiting IFN production and suppressing major histocompatibility complex (MHC) and IFN synthesis in dendritic cells (DCs) after infection [16]. One theory suggests pathogenic RABVs hinder mature DC migration to lymphoid organs, enabling them to infect peripheral nervous system (PNS) cells without a strong immune response. Virulent strains inhibit IFN release more than avirulent ones [17]. The IFN antagonist properties of RABV P, M, and N proteins are well-studied, with P protein being the most well-defined, mediated by components of the cytoskeleton, nuclear trafficking receptors, and innate immune factors [18]. P protein interacts with host proteins beyond immunity, functioning as a virus-host interface “hub” [19]. The high evolutionary flexibility in the non-enzymatic replication machinery component, encoded in all mononegaviruses via a P gene, demonstrates little to no sequence conservation within the Mononegavirales (MNV) order or within the same family [20].

### 2.1. N Protein

The N protein encapsulates the genome to protect it from nucleases, binding to its own mRNA non-specifically. It interacts with the P protein to prevent phosphorylation and eliminate non-specific RNA binding. The encapsulated genomic RNA, called the nucleocapsid (NC), functions as a coding strand for viral transcription and replication [21]. The RABV N protein acts as an IFN antagonist by acting upstream of TBK1/IRF3 at the level of RIG-I, using a different method from the P protein [22]. The pathogenic Nishigahara strain of RABV showed lower RF3 initiation and IFN/chemokine release compared to its Ni-CE derivative strain [23]. Pathogen-associated molecular pattern (PAMP) recognition may be hindered by N protein activity in encasing the rabies virus genome (replication products) by creating cage-like structures around cytoplasmic virus factories (Negri bodies) [24].

### 2.2. P Protein

The P protein of RABV plays a vital role in viral replication and immune evasion. It bridges the nucleoprotein and large protein in the RNP complex, aiding in the synthesis of viral RNA as a co-factor of RNA polymerase [25]. The rabies virus phosphoprotein has been discovered to hinder host cells’ antiviral response to type I interferon by blocking the phosphorylated form of nuclear accumulation of STAT1. This function is crucial for the virus’s pathogenicity, as the IFN-initiated response is vital for the host’s innate immunity [17]. Masatani et al. [26] studied the impact of P proteins from various RABV strains on IRF-3 signaling. They found that P proteins suppressed IRF-3-dependent IFN-β promoter activity, street strains 1088 and HCM-9 significantly reduced IFN-β promoter activity that is IRF-3-dependent and IKKe-inducible. IKKe only interacted with P proteins from these strains, and the C-terminal portion of P protein was crucial to the interaction. This suggests that RABV street strains’ P protein may help them evade the host’s innate immunity [26]. The P region, comprising aa residues 176–186, was found to be intricated in the antagonism [27]. The study compared CVS and SAD sequences of the above specific P domains, identifying two amino acid alterations at positions 177 and 179. The researchers found that replacing proline 179 with serine restored P’s ability to suppress IFN induction [28]. P1/P2 effectively excludes STATs associated with P1/P2 through an N-terminal nuclear export sequence for active nuclear export, utilizing cellular trafficking machinery [29]. N-terminal truncation activates a nuclear localization sequence (NLS) in P3, inactivates the nuclear export signal (NES), and triggers microtubule attachment [30]. Truncated P3 (tP3) is distributed to the cytoskeleton and nucleus, arresting P3-associated STAT1 and potentially blocking the interaction between STAT1 and DNA in the nucleus [31]. A study by Brzo’zka et al. [29] found that the rabies virus P protein is responsible for blocking IFN-α/β- and IFN-γ induced JAK-STAT signaling in RABV-infected cells by keeping active STATs in the cytoplasm. The study found that cells infected with RABV SAD L16 strain or cells producing RABV P protein from transfected plasmids showed significant impairments in gene expression. However, a recombinant RABV created by shifting the P gene to a distal promoter gene location (SAD ΔPLP) was unable to obstruct JAK-STAT signaling. The study also found that RABV P protein bound tyrosine-phosphorylated STATs conditionally in response to IFN activation. The 10 C-terminal residues of RABV P are necessary to block JAK-STAT signaling but not for inhibiting transcriptional activation of IFN-β [29]. RABV P inhibits STAT3 signaling similarly to STAT1 and STAT2, binding to STAT3’s final 30 amino acids at the C-terminus, preventing its accumulation in the nucleus [32]. STAT3 is implicated in Gp130-receptor-dependent pathway, triggering the initiation of additional immunological molecules, such as the cytokine family of IL-6 [33]. The inhibition of tyrosine-phosphorylated STAT3’s (pY-STAT3’s) nuclear localization results in a decrease in the IFN-stimulated cytokine response [32]. Harrison et al. [34] found that the rabies virus P protein and STAT3 interaction is dependent on STAT1, demonstrating that the P protein needs STAT1 to antagonize STAT3, suggesting specific targeting of STAT1 and STAT3-STAT1 heterodimers. They also found that STAT3’s antagonistic properties are shared by P proteins from various lethal lyssaviruses, indicating conserved roles in infection. They also explained how altering the P protein C-terminal domain’s (CTD’s) “W-hole”, the hydrophobic pocket reduces STAT3 antagonistic interactions [34]. Duvenhage lyssavirus p protein (DUVV-P) is found to be deficient in binding to STAT1, a feature similar to a (W265 on rabies virus P protein for G265 in DUVV P protein) [35]. The replacement of W265 for G and M287 for V in the “W-hole” of rabies virus or DUVV P proteins significantly reduces interferon and STAT1 binding/inhibiting and recombinant RABV pathogenesis [35]. The interaction between the P protein and STAT3 is crucial for the P protein’s interaction with the mitochondrial complex 1, as STAT3 controls the activity of mitochondria [36]. The promyelocytic leukemia (PML) promoter, which includes an interferon stimulated response element (ISRE) and an IFN-γ activation site (GAS), enables the direct generation of PML by types I and II IFNs [37]. The rabies virus caused promyelocytic leukemia and Negri bodies (PML and NBs) to rearrange, resulting in larger, dense clumps. P3 expression increases PML body size, while P cages PML in the cytoplasm. P and P3 engage in interactions with PML that involve the C-terminal region and the really interesting new gene (RING) finger. PML^−/−^ primary mouse embryonic fibroblasts produced 20 times more virus and expressed viral proteins higher, indicating a higher rate of RABV replication in the absence of all PML isoforms [38].

Viruses engage with host proteins, altering the biology of infected cells by utilizing multifunctional viral proteins. These proteins are usually viewed as collections of independent functional modules. However, this model does not explain the functions seen in certain viral proteins. For instance, the RABV P3 protein, which is a shortened version of the pathogenic P protein, showcases exclusive functions that are absent in its longer variations. A study conducted by Sethi et al. [39] examines P3 from the pathogenic RABV strain Nishigahara (Nish) and a modified strain known as Ni-CE. The study delves into the intricacies of intra-protomer interactions, highlighting the connection between the globular C-terminal domain and the intrinsically disordered regions (IDRs) of the N-terminal region. This delicate interplay leads to the dynamic transition of the fully functional Nish P3 between open and closed states. The Ni-CE P3 protein shows a notable openness, highlighting the discrete functions of this critical multifunctional viral protein depends on the conformational arrangements of distant individual domains and IDRs, as well as their individual roles. This unique conformational variation is attributed to a single mutation, N226H, in Ni-CE P3. They discovered that Nish P3 experiences liquid–liquid phase separation, while Ni-CE N226H P3 do not. This characteristics is linked to P3’s ability to engage with various cellular membrane-less organelles, especially those connected to immune evasion and pathogenesis [39].

### 2.3. M Protein

**Figure 2:**
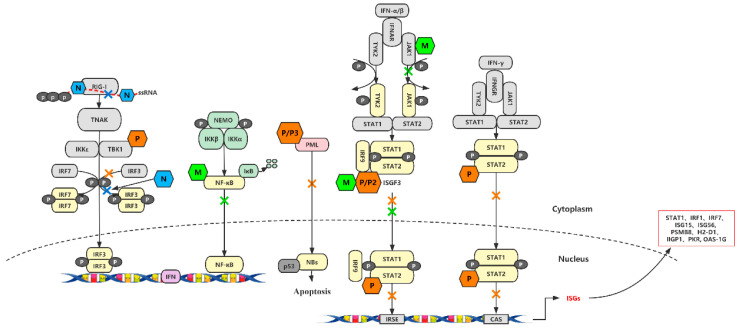
The various interactions between RABV proteins and interferon signaling pathway. The IFN-signaling pathway is activated by RABV infection, whereas RABV utilizes its own proteins to suppress the IFN signaling pathway by interacting with key factors in IFN pathways. RABV P interacts directly with tyrosine-phosphorylated STAT (pY-STAT) to inhibit the expression of ISGs (interferon-stimulated genes) by affecting its localization and reducing its ability to bind to ISG promoters. P protein is also able to interact directly with TBK-1 (TANK-binding kinase-1) in a dose-dependent manner, inhibiting phosphorylation of the IRF3 (interferon regulatory factor 3). Moreover, the interaction between P and PML (promyelocytic leukemia) alters PML protein localization and the structure of PML-NBs (nuclear bodies), thereby regulating IFN-induced apoptosis. The encapsulation of RABV RNA by N protein protects viral RNA from getting recognized by RIG-I (retinoic acid–inducible gene I), which subsequently prevents RIG-I-mediated activation of the downstream IRF-3 pathway. Type-I IFN stimulation causes the M to shift toward activated pSTAT1 interaction, which improves the ability of P protein to engage with JAK1 to prevent pSTAT1 from activating and with pSTAT1 to restrain it in the cytoplasm [43].

Lyssavirus M protein, a 20–25 kDa protein, forms oligomers for virion stability and serves as a binding platform for envelope membrane and viral glycoprotein trimers, regulating viral genome transcription and replication [40]. Novel Nuclear factor kappa B (NF-κB) RelA isoform, RelAp43, discovered in M protein screen, acts as positive regulator in NF-kB signaling and immune antagonistic activity [41]. The study found that the phosphoprotein of RABV suppresses pro-inflammatory cytokines, Type I IFNs, as well as interferon regulatory factor 1 (IRF1) and human inhibitor of apoptosis protein (HIAP), aiding in immune evasion. It also interacts with Janus kinase 1 (JAK1), shifting towards pSTAT1 interaction upon Type I IFN stimulation. The mutant M protein enhances antiviral immune responses and reduces pathogenicity in mice. This suggests that Rabies virus proteins target both NF-κB and Jak-Stat pathways to neutralize the innate immune response [42], Figure 2.

## 3. BBB Permeability and Cytokines Roles During Virulent and Avirulent RABV Infections

When infections release damage-associated molecular patterns (DAMPs) and pathogen-associated molecular patterns (PAMPs), germline pattern-recognition receptors (PRRs) recognize these conserved structures and help the innate immune system identify microbes [44]. Upon identification of viral patterns, a signal transduction triggers the release of IFNα/β, inflammatory cytokines and chemokines, in addition to luring immune cells into infected areas [45]. Cell-mediated immunity is vital for an effective immune response to combat RABV infection, with central nervous system (CNS) having innate immune cells like macrophages, peripheral immune cells like B cells and T cells, which are recruited to aid in containing and eliminating pathogens, potentially causing tissue damage [46]. Antigen-presenting cells (APCs) must trigger naïve T cells in order to initiate the adaptive immune response [47]. The CNS is an immunologically distinct site that influences the body’s response to environmental antigens. It lacks secondary lymphoid organs, it has limited lymphatic drainage and specialized antigen-presenting cells like dendritic cells (DCs), and barriers like the blood–brain barrier (BBB) restrict immune cell exchange [48].

A study by Roy A and Hooper [49] found that SHBRV infection triggers robust immune response in the host’s peripheral nervous system. However, failing to breach the BBB and transfer of immune effectors to CNS tissues often leads to death. Rabies virus infection is less likely to be fatal in mice with a PLSJL background, which has decreased the activity of the hypothalamic pituitary adrenal axis (HPA) and enhanced the ability to modulate inflammatory responses. Half of PLSJL mice survive SHBRV infection, due to their ability to mediate BBB permeability alterations [49].

In 2008, Roy et al. [50] conducted a study that examined the evolution of immunity in the peripheral and central nervous systems in mice infected with different pathogenic strains of RABV. In their investigation, they detected only one strain, HEP, that does not seem to have moved to the 129/SvEv mice’s central nervous system [50]. In the central nervous system, tissues from mice inoculated with HEP, which is thought to be less neuro-invasive because it has glutamine at position 333 in its glycoprotein. They were not able to identify viral antigen or signs of innate immunity to the virus [51]. Meanwhile, other RABV strains were found to be neuro-invasive in mice. Some strains, like challenge virus standard F3 (CVS-F3), Evelyne–RoKitnicki–Abelseth (ERA) and Pitman–Moore (PM) strain, allowed the mice to survive, but others, like dog rabies virus (DRV-4), Thailand genotype 1 dog lyssavirus (Thai-DRV), as well as Skunk RABV, were fatal to 129/SvEv mice. The authors categorized RABV into three classes: poorly neuro-invasive viruses, neuro-invasive viruses eliminated by immune response, and lethal viruses that do not exhibit changes in BBB permeability. Lymphocytes from mice infected with lethal RABV variants were able to clear CVS-F3, suggesting a potential treatment option. Immunocompromised recipients may benefit from the adoptive transfer of lymphocytes derived from mice inoculated with variants of RABV [52]. Thus, scientists have deduced from these tests that there is a component of the SHBRV infection that prevents immune effectors from penetrating central nervous system (CNS) regions [52]. It was shown that LSJL mice, which display a more robust CNS inflammatory response, had an increased ability to eliminate SHBRV [49]. The study examined RABV strains’ infectivity in PLSJL and 129/SvEv mice, aiming to determine if any host response component is involved in RABV infection, as demonstrated in SHBRV [49]. PLSJL mice showed reduced pathogenicity for DRV-Mexico, coyote street rabies virus (CosRV), and Skunk RABV strains, while Thailand genotype 1 dog lyssavirus (Thai-DRV) and CVS-N2c RABV strains were highly deadly to PLSJL and 129/SvEv mice. The mortality of RABV infection is influenced by viral and host characteristics. CVS-N2c’s N protein mRNA was found in the central nervous system of inoculated 129/SvEv as well as PLSJL mice, while CosRV nucleoprotein mRNA was found in lethally infected CNS [49].

Chai et al. [53] examined the induction of tight junction (TJ) proteins in the central nervous system after intracerebral injection with laboratory-fixed CVS-B2c or virulent RABV DRV-4 to determine the process rabies virus infection promotes blood–brain barrier permeability. Mice infected with laboratory-attenuated RABV showed considerably increased BBB permeability, but not with wild-type RABV. Mice inoculated by laboratory-fixed RABV showed lower expression of TJ proteins (claudin-5, occludin, and zonula occludens-1) than mice inoculated with wild-type RABV. This suggests that increased BBB permeability is associated with decreased TJ protein expression in RABV infection. Neither infection nor alteration of TJ protein release in brain microvascular endothelial cells (BMECs) is caused by RABV [53]. IFN-γ, not TNF-α, increases BBB permeability in RABV infection. Neutralizing IFN-γ can reduce BBB permeability in mice and restore TJ protein release in brain microvascular endothelial cells (BMECs) infected with laboratory-attenuated RABV [54]. These findings indicate that wt RABV infection does not directly cause BBB permeability, but viral-induced chemokines/cytokines do.

**Figure 3:**
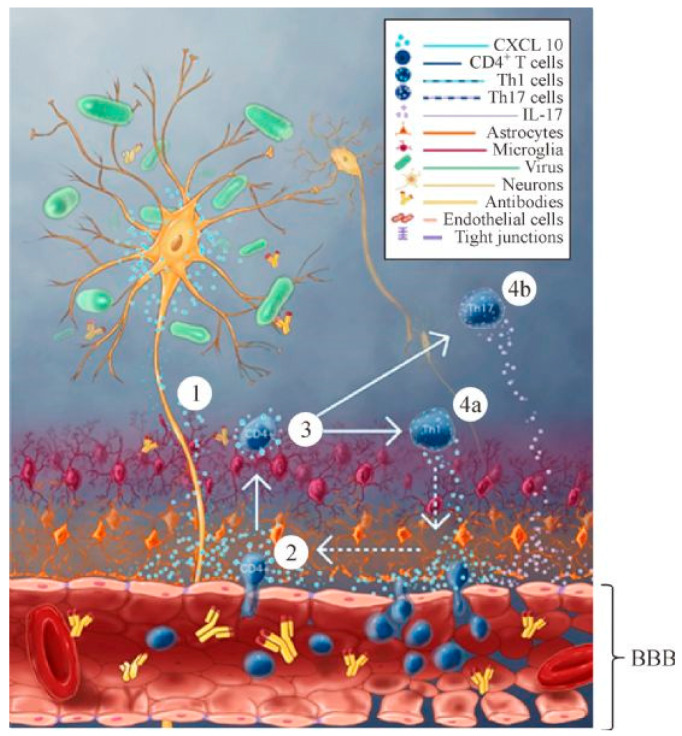
The method by which neural production of CXCL10 caused by laboratory-attenuated rabies virus (RABV) enhances the blood–brain barrier (BBB) permeability. 1. Chemokine CXCL10 is secreted by laboratory-attenuated RABV infected neurons; 2. CXCL10 mediates CD4^+^ T cell recruitment into the CNS; 3. CXCL10 mediates CD4^+^ T cell differentiation into Th1 and Th17 cells; 4a, IFN-γ secreting Th1 cells could further boost the induction of CXCL10 through positive feedback; 4b, IL-17 secreting Th17 cells alters the TJ (tight junction) proteins leading to Breakdown of the blood–brain barrier [67].

In a related study conducted by Chai et al. [55], mice were inoculated by either the virulent RABV strain, DRV-4, or the laboratory-fixed RABV strain, CVS-B2c, in order to ascertain the spatiotemporal comprehensive expression of CXCL10 in rabies virus infection. Neurons, astrocytes, and microglial cells are examples of resident brain cells that can release CXCL10. Immune cells that move in from the periphery can also produce it [55]. Research on the relationship between time and space revealed that neurons were the first to exhibit CXCL10 expression. The IFN-γ inducible CXCL10 interacts with the CXCR3 receptor, which is expressed on activated CD4^+^ cells and controls lymphocyte movement to the central nervous system [53]. CXCL10 stimulates the migration of CXCR3^+^ CD4^+^ T cells into the central nervous system, transforming them into Th17 cells producing IL-17 and Th1 cells releasing IFN-γ [56]. During RABV infection, the Th1 cells secrete IFN-γ, then they augment CXCL10 release and enhance positive feedback. CXCR3^+^ positive CD4 cells then move to the central nervous system [53], Figure 3. The CNS Th17 cells’ production of IL-17 starts to change the tight junction (TJ) proteins [57] Figure 3. The study discovered that anti CXCL10 antibody therapy inhibited CXCL10, which reduced the expression of IL-17 and IFN-γ, thereby reducing BBB permeability and Th17 cell infiltration to the brain, while also restoring TJ protein expression and BBB integrity in RABV infection [55]. Neuronal CXCL10 triggers microglia and astrocyte activation, inflammatory cell infiltration, chemokine/cytokine expression, decreased TJ protein, and improved BBB permeability. Similar to rabies infection, depleting CXCL10 reduces CXCR3^+^ positive CD8^+^ cells invasion during infection by West Nile virus (WNV) [58].

Administering monocyte chemoattractant protein-1 (MCP-1) after virus-neutralizing antibodies (VNA) treatment improved survival rates to 80%, but VNA administered peripherally did not clear rabies in CNS [59]. The study found that modifying the permeability of the BBB is crucial for surviving RABV infection. Once rabies virus enters the CNS, virus-neutralizing antibodies (VNAs) in the peripheral nervous system are not able to infiltrate the blood–brain barrier and enter the central nervous system. It is suggested that virus-neutralizing antibodies (VNAs) released within the central nervous system by invasive B cells are essential for removing RABV. Experiments in mice showed that maintaining elevated BBB permeability and injecting VNA can eliminate RABV and prevent rabies formation. This research lays the foundation for potential VNA treatment in human clinical rabies [59]. Currently, post exposure prophylaxis (PEP) mandates the passive delivery of rabies virus VNA [60]. While VNA may not work once the virus infects CNS tissues, they likely hinder its further spread. However, using passive antibody administration to target CNS mechanisms promoting immune delivery would contradict this, as mice vaccinated with the deactivated rabies vaccine showed strong levels of virus-neutralizing antibodies. Nonetheless, they offered limited protection against an intracranial virus challenge compared to intramuscular administration [61]. Combining live-attenuated and inactivated vaccines is vital for eradicating rabies virus from CNS [61]. The outcome of a wild-type RABV infection in the CNS depends on the degree of infection and whether immune clearance results in significant neuron loss.

It is important to note that, due to the low fidelity of viral RNA polymerase, it is crucial to be aware of the possibility of reversal during virus passage in infected cells. New research shows negative-sense RNA viruses can undergo homologous recombination [62]. Efforts to manipulate the rabies virus genome by employing reverse genetics are crucial to producing effective vaccines and gene products. Careful planning and design are necessary for the development of future rabies vaccines, ensuring a balance between immunogenicity and biosafety. Live-attenuated viruses have shown promise as vaccines for diseases like measles, smallpox, and yellow fever. While the use of live-attenuated RABV strains is limited to animals, obtaining highly attenuated strains with increased immunogenicity through reverse genetic techniques could provide a new approach to develop vaccines for rabies and other viral pathogens [63].

To investigate their involvement in rabies, chemokine genes, including macrophage-derived chemokine (MDC), CCL5, CXCL10, and MIP-1a, were introduced into the RABV genome. Recombinant rabies virus releasing CXCL10 or CCL5 cause continued production of these chemokines, leading to inflammation in the central nervous system, disease progression, and death. Recombinant RABVs expressing macrophage inflammatory protein-1α (MIP-1a), macrophage-derived chemokine (MDC), and granulocyte monocyte-colony stimulating factor (GM-CSF) enhance blood–brain barrier permeability, temporarily produce chemokines, and attract inflammatory cells. These recombinant RABVs also stimulate immune reactions by activating B cells, T cells and dendritic cells. They can even prevent rabies infection when injected into the CNS. This suggests that chemokines play both harmful and protective roles in RABV infections, potentially guiding the development of future vaccines or treatments [64].

The lethal nature of rabies and similar lyssavirus infections is due to the virus entry into the CNS. Current immunotherapies aim to neutralize the virus before symptoms appear. A latest investigation by Mastraccio et al. [65] evaluated the effectiveness of F11, a human monoclonal antibody, in treating chronic lyssaviruses infection. One dose of F11 reduces the virus titer in the brain and then reverses clinical symptoms after lyssavirus infection, even when given after virus replication begins. An adaptive immune response relying on CD4 T cells is crucial for effective infection management, as F11 dependent neutralization alone is insufficient for survival. F11 alters leukocyte populations in the brain and its binding to Fc receptor (FcRc) enhances treatment effectiveness.

In a previous study related to the antibody-based treatment potential of symptomatic rabies, Guilherme Dias de Melo et al. [66] reported that merging dual robust neutralizing human’s monoclonal Abs given against the virus envelope glycoprotein cures rabid mice that were already symptomatic. Antibodies must be given intracerebroventricularly into the CNS and simultaneously injected peripherally for the treatment to be effective. After receiving such care, the mice were healed and, showed excellent clinical condition, viral loads were not detectable, and displayed nearly normal brain inflammatory profiles [66]. Their investigation offers a crucial practical potential of an Ab-based remedial approach for rabies patients who exhibit symptoms.

## 4. Apoptosis

Inducing apoptosis can halt virus spread and is considered among the nonspecific primary defense mechanism of the body. Some viruses hinder cell apoptosis to escape immune responses [68], allowing them to continue replicating inside the cells, while other viruses multiply quickly enough to spread before the cell goes through apoptosis [69].

The extrinsic and intrinsic routes are the two primary pathways in the mechanism of apoptosis [70]. Extrinsic pathways are through the death receptor, while intrinsic pathways are mitochondrial-mediated. Death receptors, such as tumor necrosis factor receptor type 1 (TNFR1), Fas, and tumor necrosis factor-related apoptosis-inducing ligand (TRAIL) receptors, activate the extrinsic pathway when attached to TNF, Fas-L, and factor-related apoptosis-inducing ligand (TRAIL) [70]. The entrance of Bcl-2-associated X protein/Bcl-2 antagonist killer 1 (Bax/Bak) into the mitochondrial membrane initiates the intrinsic apoptotic pathway. This leads to the expression of cytochrome c (Cyt c), forming an apoptosome with procaspase-9 and apoptotic protease activating factor-1 (Apaf-1). Then, the activation of the caspase 3 cascade ensues.

The Fas protein belongs to the TNF-α receptor family and has a death domain in the cytoplasm [71]. Fas ligand (FasL) is expressed by a few cell types, including those in immune-privileged areas like the central nervous system, and Fas-bearing cells undergo rapid, caspase-dependent apoptosis [72]. A study by Lafon [73] showed that acute RABV infected mice’s central nervous systems express higher levels of Fas ligand (FasL), which kills activated migratory T cells, supporting the idea that FasL overexpression is crucial for RABV pathophysiology.

B7-CD28 group’s Programmed death-ligand 1 (PD-1) inhibitory molecule, B7 homolog 1 (B7-H1), binds to PD-1, inhibiting T cell multiplication and cytokine release, potentially contributing to RABV immune-evasion [74]. Pathogenic RABV can eliminate T cells by exploiting the host’s immunosuppressive systems, as evidenced by milder symptoms in B7-H1 knockout mice in contrast to wild-type mice. The study found that B7-H1 knockout mice had lower apoptotic CD8^+^ cell proportions in the CNS, and greater percentages of CD8^+^ and CD3^+^ T cells in the overall T cell infiltration compared to the wild-type mice’s CNS [75]. The study indicated that T cells invading the central nervous system undergo B7-H1-mediated apoptosis, a vital role in RABV pathophysiology and inflammatory response inhibition. Neurons express B7 homolog 1 (B7-H1), human leukocyte antigen-G (HLA-G), and Fas ligand (FasL), which interact with T cell receptors, promoting viral invasion [76].

The gaseous, inorganic free radical nitric oxide (NO) exhibits pleiotropic characteristics and is converted to NO by nitric oxide synthase from L-arginine in the presence of O_2_ and NADPH [77]. Inflammatory phagocytes, epithelial cells, and neurons express inducible nitric oxide synthase (iNOS), which produces more nitric oxide for extended periods (10–100 times more) [78,79]. Primarily nitric oxide is generated in the brain, by natural killer (NK) cells and macrophages and it has been linked to neurodegenerative illnesses like rabies [80]. Madhu et al. [81] investigated the NO’s role in controlling immune responses due to rabies virus infection. They injected mice with CVS strain and amino-guanidine (INOS inhibitor) and found that CVS-infected mice exhibited severe symptoms and shorter survival, while CVS and aminoguanidine (AG) treated mice showed milder symptoms and longer onset time. Serum nitrate levels were higher in CVS-infected mice [82]. Comparing to mice treated with CVS-AG, the CVS-infected group showed reduced natural killer, CD4^+^ and CD8^+^ cells in blood and spleen at 4 dpi. [82]. NO inhibition reduced rabies viruses, apoptotic molecules, caspase 1, as well as FasL expression in the brain [81].

NO-induced apoptosis plays a critical role in the aggravation of rabies virus infection and immune response control. Lower levels of caspase 1, FasL, and other apoptosis molecules may result in increased levels of CD4^+^ T cells, CD8^+^ T cells, and NK cells within the CVS-AG treated group. RABV successfully invades the nervous system by preventing T lymphocytes from infiltrating the CNS and evading virus-induced early apoptosis.

## 5. Autophagy

Autophagy is a cellular regulation mechanism that transports unhealthy cytoplasmic material to the lysosome for destruction [83]. Different types of autophagy include micro, macro, and chaperones mediated autophagy. Macroautophagy involves phagophores encasing substrates to form vesicles [84]. Autophagy is triggered by heat shock, energy stress, hypoxia, and infections [85]. Autophagy helps eliminate intracellular pathogens, and contributes to both innate and adaptive immunity, including presenting antigens. [86].

The pathogenic strain CVS-11 triggers incomplete autophagy in NA cells [87], discovering a link between BECN-1(BECN1) and CASP2 in the autophagy route, with RABV inducing incomplete autophagy through BECN1’s binding to the viral protein P. Controversy surrounds whether the HEP-Flury strain induces incomplete autophagy in NA cells due to varying strains and cells used in different labs.

Liu et al.’s 2020 study [88] found that P5 reduces BECN1-dependent caspase 2 (CASP2) levels and increases AMPK, MAPK, AKT, and MTOR phosphorylation. They found that RABV infection in N2a cells was downregulated when Becn1 was silenced using two siRNAs. This suggests that P5 plays a beneficial role in regulating RABV infection, which is dependent on BECN1. Incomplete autophagy is caused by the P protein’s amino acid residues 173–222 [88].

## 6. Mitochondrial Dysfunction

Mitochondria are vital organelles that regulate the programmed cell death process, releasing pro-apoptotic factors after losing their membrane potential due to apoptotic signals [89]. Twenty-seven Bcl-2 family proteins, regulated during apoptosis, depend on pro-apoptic proteins Bcl-2 antagonist killer and or Bcl-2-associated X protein 1 (Bak and/or Bax) for mitochondrial triggering [90]. When a Bcl-2-associated X protein (Bax) is activated, it changes shape, enters mitochondria, forms oligomers, and loses membrane construction ability [91]. Bax, an inactive monomer in healthy cells’ cytoplasm, is inhibited by Bcl-2, an anti-apoptotic protein [92]. Bcl-2 homology domain 3 (BH3)-only proteins, including BCL2 associated agonist of cell death (Bad), are crucial for triggering apoptosis by transmitting signals to other Bcl-2 family members and releasing pro-apoptosis proteins, such as Cyt c and AIF [93]. The mitochondrial caspase-dependent apoptotic pathway, represented by Cyt c, activates caspase-9, leading to irreversible cell death. Apoptosis-inducing factor (AIF) is cleaved and truncated, causing DNA breakage and chromatin condensation in the nucleus, known as caspase-independent cell death [94]. Many viruses control cell apoptosis at the mitochondrial level, where pro- and anti-apoptotic signals are combined [95].

### 6.1. Role of RABV Proteins

Through its interaction with Complex I, the rabies virus P protein plays a crucial role in the production of mitochondrial malfunction and formation of reactive oxygen species, leading to oxidative stress in RABV infection [96]. A peptide with amino acids 139–172 of P boosted ROS generation and Complex I activity, similar to the full P protein [96]. Mitochondrial malfunction causes a deadly condition with neuronal degeneration and oxidative stress [96]. Proteomics evaluated mouse neuroblastoma cell mitochondria extract enriched with P and peptides from RABV G, P, and N was also detected [97]. The connection between the RABV P and complex I causes RABV infection, a mitochondrial disease; crucial locations for this interaction in the P are S162 and S166 [98]. Mitochondrial malfunction causes severe clinical conditions, oxidative stress in neurons, and degenerative alterations, impacting neuronal processes. This knowledge aids future rabies treatment creation [98]. STAT3, regulates P protein’s interactions with mitochondrial complex I and II [36]. The study indicated that the RABV P protein inhibits STAT3 signaling, leading to a reduction in complex I activity when STAT3 is blocked [32].

Attenuated ERA strain induces more apoptosis and G proteins than CVS [99]. The virulence of RABV negatively correlates with apoptosis, while the expression level of the G protein affects the RABV’s ability to induce cell death [100]. Li et al. [101] modified the wild-type RABV SAD strain by replacing G residues and found that the RABV SAD strain with G (K83R) mutation was less virulent, causing more apoptosis in infected cells.

Liu and colleagues [102] found that silencing or deactivating G Protein-Coupled Receptor 17 (GPR17) promotes RABV replication in N2a cells, while overexpressing or activating GPR17 decreases it. Overexpression promotes RABV-induced apoptosis, linked to Bcl-2 antagonist killer 1 (BAK), which is inhibited by BAK knockdown, implying GPR17 promotes BAK-mediated intrinsic apoptosis.

The cleavage of caspase-3 and apoptosis are reduced when caspase-9 activation is blocked instead of caspase-8 [103]. The mitochondrial pathway is primarily responsible for CVS infection apoptosis, with the Bcl-2 family being the primary modulator. CVS infection initially prevents apoptosis by downregulating Bcl-2-associated X protein (BAX) expression, but later induces it by upregulating apoptosis-inducing factor (AIF) and boosting cytochrome c release [104]. N2a cells undergo caspase-dependent and caspase-independent apoptosis, while chimeric recombinant viruses with wt RABV M gene can cause human neuroblastoma cell line (SK) and NA cells to convert from microtubule linked LC3-I to LC3-II [105]. RABV induces mitochondrial death in late replication, controlled by the BCL-2 gene family. Apoptosis and RABV proteins M, G, and P, are intricately linked. Cytochrome c and AIF factors are released when mitochondrial apoptosis is triggered by M protein inhibition of Bcl2. Apoptosis can be induced directly by released AIF.

Tian et al. [106] conducted an experiment using NA cells and Chimeric RABVs with single GD-SH-01 gene in the HEP-Flury genome using a reverse genetics approach in order to determine the causes behind the phenotypic variations between wt GD-SH-01 and attenuated HEP-Flury (isolated from a rabid pig) [107]. Researchers have found a connection between the P gene of rabies virus and host cell death following viral infection. They also created rRABV, and rGDSH-P, the P gene from HEP-Flury, reveals that P gene activates the intrinsic arm of the apoptotic pathway. Infections with rHEP-shP or GD-SH-01 cause caspase-9 and caspase-3 activation, mitochondrial cytochrome c release, bcl-2 downregulation, mitochondrial membrane potential (MMP) decrease, and poly-ADP ribose polymerase (PARP) cleavage.

ERA G protein induces apoptosis, unlike N protein; ERA G protein is more effective than CVS G protein [99]. Human cells were infected with a recombinant strain of rabies virus, replacing the G protein gene with a more harmful version. Only the strain carrying the G protein caused apoptosis in cells, showing its significant role in triggering apoptosis [99].

Kassis et al. [108] conducted a study on lyssaviruses, infecting cells with Mokola virus (MOK), Thailand virus (THA), and Lagos bat virus (LAG). They found that a TRAIL-dependent process involving caspase-8 initiation that leads to apoptosis in neuroblastoma cells infected with MOK and LAG. Caspase-3 and caspase-6 activated, but caspase-1 and caspase-2 showed no activity. Lyssavirus replication and decapsulation are necessary for apoptosis induction, with TNF-α and Fas ligand playing a minor role [108]. This suggests that virus initiation of apoptosis could probably be multigenic but not merely linked to the virus glycoprotein [109]. Delayed apoptosis noted in THA, leading to more severe pathophysiology compared to MOK or LAG [108].

**Figure 4:**
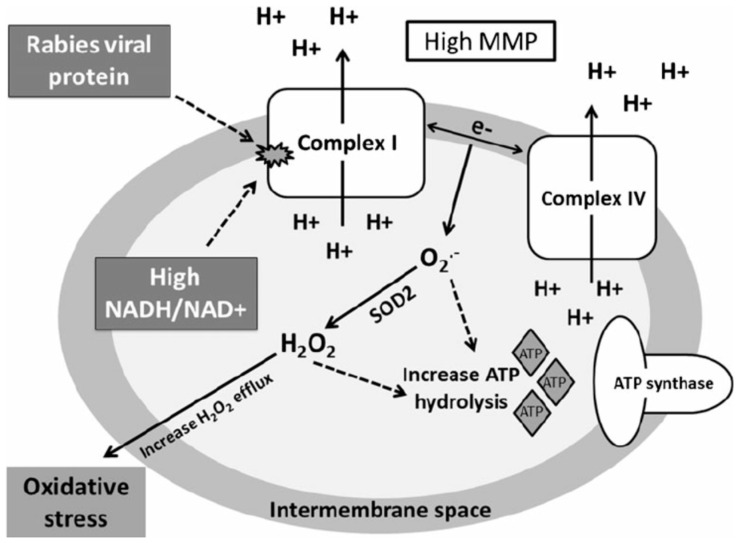
Mechanisms of mitochondrial dysfunction associated with rabies virus infection. In mitochondria of rabies virus-infected neurons, the activity of electron transport system (ETS) complexes I and IV are increased either due to direct (e.g., interaction with a rabies virus protein) and/or indirect (e.g., high NADH/NAD^+^ ratio and up-regulation of sirtuin activity) viral effect. The increased proton pumping across mitochondrial membranes generates a high mitochondrial membrane potential (MMP). Electron leakage during both forward and reverse ETS leads to superoxide (O_2_^−^) formation that is dismutated by mitochondrial superoxide dismutase (SOD_2_) to hydrogen peroxide (H_2_O_2_). Both O_2_^−^ and H_2_O_2_ may lower the intracellular ATP levels by increasing hydrolysis of ATP molecules. Overproduction and accumulation of O_2_^−^ and H_2_O_2_ induces oxidative stress and leads to degeneration of neuronal processes (solid lines findings, dashed lines hypothesis) [110].

A study by Alandijany et al. [110] used dorsal root ganglion (DRG) cells, pheochromocytoma (cancer) cells (PC12) and mouse neuroblastoma cell line (MNA) to examine the effect of CVS-11 strain on mitochondrial dysfunction. They found that all three cell lines showed increased activity in complexes I and IV, but not complexes II and III. PC12 and MNA cells also exhibited a marked increase in mitochondrial membrane potential. Additionally, the intracellular ATP levels of CVS-treated PC12 and MNA cells were considerably less as compared to the mock-infected cells 72 h after infection. Nicotinamide adenine dinucleotide (NAD) + hydrogen (H) (NADH/NAD^+^) ratio in CVS-infected PC12 cells was higher, indicating a change in the redox state. Hydrogen peroxide (H_2_O_2_) detection showed similar levels of ROS production in CVS-treated MNA cells as compared to mock-infected cells after 72 h of infection [110] Figure 4.

In vitro studies showed that rabies virus damages dorsal root ganglion (DRG) neuron axons through oxidative stress, implicating mitochondria in neuro-degeneration. In vivo studies with infected mice reveal axonal degeneration from decreased axonal transport [111]. Oxidative stress contributes to axonal swelling and degeneration seen in experimental and natural rabies in humans and animals.

Larrous et al. [112] evaluated M proteins from two viruses (THA and MOK) with varying apoptosis-causing abilities. A 20 aa segment (at locations 67 to 86) inhibits cytochrome c oxidase and induces apoptosis as MOK’s M protein [112]. Activation of cell death pathways depends on aa at locations 77 and 81, with position 77 influencing cytochrome c oxidase (CcO) activity and position 81 affecting factor related apoptosis inducing ligand (TRAIL)-dependent apoptosis. Mutations impairing pathway induction delay apoptosis compared to the non-mutated control [112]. CcO1 and CcO2 form the enzyme’s catalytic core, facilitating electron transfer from cytochrome c to oxygen to produce water. This generates a proton motive force that drives adenine triphosphate (ATP) synthesis [113]. The majority of genotype 1 strains poses R as well as E, at sites 77 and 81, while genotype 2 as well as genotype 3 contains K and N at the same positions [114]. Genotype 1 residues R77 and E81 increase pathogenicity potential [112].

CVS-B2c and CVS-N2c are stable rabies virus strains with significant differences in pathogenicity and non-neuronal cell infectivity. The glycoprotein (G protein) is implicated in the virus’s pathogenicity, but both CVS-B2c and CVS-N2c have no difference on the antigenic site III, unlike previous research [115]. CVS-B2c, a less virulent strain, induces a minimum of fourfold higher G protein levels compared to CVS-N2c in infected neuron cells, suggesting that pathogenicity is inversely related to G protein expression levels, with post-translational processes influencing G protein stability playing a major role [115]. Pulse-chase studies showed that CVS-B2c’s G protein degrades slower than CVS-N2c’s, leading to programmed cell death in neurons infected with CVS-B2c. Nucleoprotein (N protein) expression levels are similar, but transport of nucleoprotein is repressed in CVS-B2c-infected cells. Attenuation of G protein release backs to rabies virus pathogenesis by averting apoptosis [115]. Apoptosis has significantly reduced in CVS-N2c-treated neural cells, in which, glycoprotein (G) level was the least.

The morphological changes observed in moribund CVS-11 infected mice differ from the one described by Li and colleagues [116]. A study by Scott et al. [111] assessed the effects of RABV infection on neuron structure using experimentally infected genetically modified mice expressing yellow fluorescent protein (YFP). The study found degeneration and disarray of pical dendrites in hippocampal regions in mice inoculated with CVS-N2C strain, while hippocampus neurons in YFP mice displayed few structural abnormalities. A few mitochondria were still visible, but Li et al. [116] also noted internal organelles, like rough endoplasmic reticulum and free ribosomes, vanished almost completely. Golgi apparatus and mitochondria grew larger. Other organelles remained constant, despite some reduction in rough endoplasmic reticulum. Scott et al.’s study [111] suggested that intracerebral inoculation leads to more severe pathological alterations than peripheral routes, possibly due to the CVS-N2C strain’s higher pathogenicity compared to CVS-11 strain.

The belief that neuronal dysfunction, not morphological changes, leads to the symptoms and death from rabies virus infection is challenged by observed structural changes in yellow fluorescent protein (YFP) mice. While traditional rabies models showed minimal neurologic changes, this study found significant alterations to organelles and neuronal processes, suggesting these changes may explain the severity of the disease.

Yuan et al. [117] explored how lyssavirus causes neuron degeneration by binding to Solute Carrier Family 25 Member 4 (Slc25a4) protein during RABV infection. They found that the virus’s M protein impairs mitochondrial metabolism, leading to decreased Nicotinamide adenine dinucleotide (NAD^+^) synthesis and increased Ca^2+^ entering the cytoplasm, activating calpains that break down α-tubulin. The study identified that the dog-derived rabies virus strain (DRV) M protein cannot break down α-tubulin, and a mutation in the 57th amino acid lessens α-tubulin degradation, reducing axonal degeneration [117].

### 6.2. Role of Nitric Oxide

Nitric oxide (NO) is the smallest bioactive molecule generated by nitric oxide synthase enzymes impacting neurotransmission, vascular function, defense, and immunity [118]. Three nitric oxide synthase (NOS) isoforms have been identified: inducible nitric oxide synthase (iNOS) or NOS2, neuronal nitric oxide synthase (nNOS) or NOS1, and endothelial nitric oxide synthase (eNOS) or NOS3 [119]. Mitochondrial nitric oxide synthase (mtNOS) is another recently discovered and little studied enzyme. Although mtNOS does not react with eNOS or nNOS antibodies, it is similar to the constitutive NOS isoforms in terms of Ca++ sensitivity and constitutive expression. Rather, it strongly bound calmodulin and displayed iNOS-like immunoreactivity and molecular weight (125–130 kDa), indicating that mtNOS is related to iNOS [120]. Neural and epithelial cells are the primary sites of expression for the calcium-dependent enzymes nNOS/NOS1 and eNOS/NOS3, respectively. A range of cells can produce calcium-independent iNOS/NOS2 when activated by cytokines, impacting the immune system as a pro-inflammatory mediator. Nitric oxide (NO) plays dual roles in immune responses, eradicating microbes but also causing pathogenic consequences by damaging cells, contributing to disorders like Borna disease [121]. The main cells involved in NO’s antimicrobial effects are macrophages, neutrophils, monocytes, and endothelial cells [122]. iNOS regulates immune cell development and function through nitration of key molecules. Expressed in T cells, macrophages, and myeloid dendritic cell (mDCs) [123].

Nitric oxide may contribute to neurological illnesses like rabies, as it interacts with superoxide to create peroxynitrate, damaging the blood–brain barrier through lipid peroxidation [80]. Regulates immune response by limiting T cell multiplication in the body [82]. NO concentrations affect T cell response, with elevated concentrations inhibiting immune system and disrupting IL-2R signaling by inhibiting the activation of Janus kinases and STAT5 [124,125]. They activate apoptosis in T cells by enhancing the expression of p53, caspase, and Fas ligands [126], or by controlling the stimulation of Bcl-2 members [77]. Low NO levels stimulate Th1 (CD4^+^ and CD8^+^ T cell) differentiation but not Th2 [127]. It has been demonstrated that low-to-moderate NO levels shield T cells from apoptosis [82].

A study by Phares et al. [54] found that BBB permeability in mice remains unchanged in the absence of TNF-α, whether it is related to CNS autoimmunity or rabies virus clearance. The absence of T cells did not affect BBB integrity when TNF-α production increased due to RABV infection. CD4 cells are necessary for increased blood–brain barrier permeability in CNS infection. Peroxynitrite (ONOO^−^) and IFN-α play roles in mediating BBB permeability during CNS infection and virus clearance without causing clinical consequences [54]. Wei et al.’s [128] study on rat cerebellar granule cells treated with NO found that intracellular ROS buildup coincides with neuronal death caused by Soluble N-ethylmaleimide-Sensitive Factor Attachment Proteins (SNAP), supporting the theory of oxidative stress as a key process [128]. In clinical situations, mitochondria produce endogenous ROS primarily [129]. Flow cytometry with 5.7.2 Dihydrorhodamine 123 (DHR123) probe found that SNAP exposure increased mitochondrial ROS levels within three hours. Intracellular ROS levels peaked six hours after SNAP administration, according to the 2′−7′ dichloro-dihydro-fluorescein diacetate (DCFH-DA) probe result. In most cases, blocking the electron transport route causes mitochondria to produce ROS [129].

Neuronal cell mitochondrial respiratory chain enzymes (Complex I–IV) are inhibited by nitric oxide, according to studies [130]. The restriction of mitochondrial respiration by nitric oxide may boost electron leakage and generate endogenous reactive oxygen species (ROS), primarily O_2_^−^, as detected in particles that are sub-mitochondrial [131]. Superoxide anion, a stable oxygen free radical, diffuses across biological membranes and undergoes non-enzymatic and enzymatic reactions to transform into hydrogen peroxide. NO can increase hydrogen peroxide’s cytotoxicity, according to reports [132]. Peroxynitrite is a harmful byproduct of high-rate interaction between NO and superoxide. Peroxynitrite and hydrogen peroxide are highly reactive and harmful, causing oxidative damage and apoptosis [133].

NO is linked to neurological disorders, with endogenous ROS production being a key factor in NO-induced neurotoxicity. Antioxidants like Potassium ascorbyl-tocopheryl phosphate (EPC-K1), which can scavenge ROS, could prevent neuronal cell death, and potentially be used as future medications for NO^−^ related nervous system disorders. EPC-K1 has been shown to enhance rat brain antioxidant capacity [134]. EPC-K1 pre-treatment significantly reduces NO-induced apoptotic and oxidative stress in cells, suggesting potential use as a medication for conditions linked to overproduction of NO.

**Figure 5:**
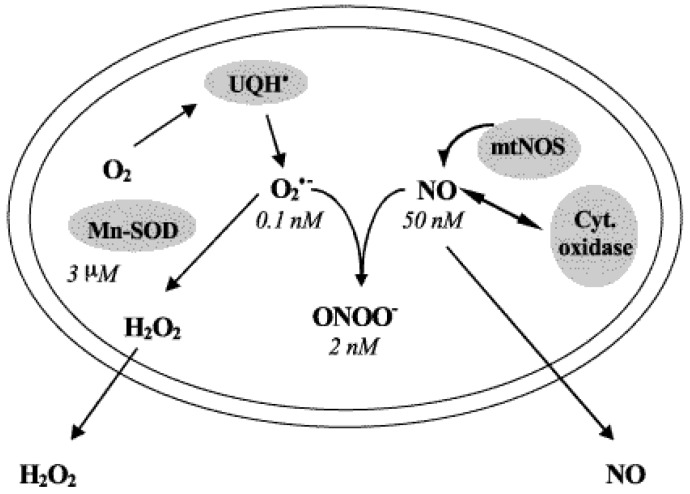
Intramitochondrial metabolism of nitric oxide and superoxide radicals. The steady-state concentrations are indicated. Oxygen and nitrogen-reactive species are kept in biological systems at steady-state concentrations that can be estimated by using the steady-state approach with the assumption that the rate of production is equal to the rate of utilization. The primary production of O_2_^−^, cytosolic Cu-Zn-SOD and mitochondrial Mn-SOD keep steady-state concentrations of 10^−10^ M in the mitochondrial matrix and 10^−11^ M in the cytosol. The cytosolic steady-state concentration of H_2_O_2_ estimated from the rate of H_2_O_2_ generation by subcellular sources and its removal by catalase and glutathione peroxidase is about 10^−7^–10^−8^ M. considering the rate of H_2_O_2_ production, its removal by intra-mitochondrial glutathione peroxidase and its diffusion to the cytosolic space, H_2_O_2_ steady-state concentration in the mitochondrial matrix results in approximately 10^−8^ M. The balance between NO production and its utilization by the reactions with the components of the respiratory chain and with O_2_^−^ regulates the intra-mitochondrial steady-state concentration of NO at about 50 nM, which in turn regulates mitochondrial oxygen uptake and energy supply [137].

At low levels, NO inhibits O_2_ absorption in the mitochondria. At 50–100 nM, it hampers cytochrome oxidase activity. NO also disrupts electron transport between cytochromes b and C1at 0.3–0.5 μM [128]. Furthermore, exposing rat liver or skeletal muscle mitochondria to nitric oxide significantly speeds up the speed at which O_2_^−^ as well as H_2_O_2_ are produced [131]. Most mitochondrial NO is utilized to produce O_2_^−^ and H_2_O_2_ at physiological NO concentrations of 20–50 nM [135]. Inhibiting complex III and cytochrome c oxidase, along with direct interactions between NO and membrane ubiquinol, raise ubiquinone’s (UQ’s) concentration. Higher NO concentrations lead to rapid reaction with O_2_^−^, generating peroxinitrite (ONOO^−^), a potent oxidant and nitrating agent [136]. NO production regulates matrix steady-state peroxinitrite (ONOO^−^) concentration and cytochrome c oxidase activity. Low ONOO^−^ yields are detoxified by reactions with mitochondrial components for instance, ubiquinol or NADH [137] Figure 5. The presence of a tiny amount of nitrotyrosine in the normal organelles is indicative of a steady, low ONOO^−^ concentration [138]. High NO concentrations cause protein and lipid oxidation/nitration, impairing mitochondrial functioning. Fast proteolytic degradation primarily targets oxidized and nitrated proteins, with new data suggesting both nitrated and denitrated mitochondria [139]. Protein nitration is a selective, dynamic, and reversible process that can be both necessary and harmful. It is often seen as a cumulative and damaging process, where nitro-tyrosine containing proteins lose function and impair cell function. The role of O_2_^−^ in the creation of ONOO^−^ or conversion to H_2_O_2_ depends on NO concentrations and reaction rates. At low NO concentrations, O_2_^−^ should be dismutated to H_2_O_2_, but at higher NO levels, ONOO^−^ production is favored. Additionally, ubiquinol (UQH_2_) and ONOO^−^ can react to produce more O_2_^−^ [136]. The process of detoxifying mitochondria from long-lasting NO effects results in the production of nitrite, nitrate, and H_2_O_2_ through ubiquinol-centered processes.

In the mitochondrial nitric oxide pathways, NO is used in three primary processes that produce temporally reversible NO activity. The three reactions that NO participates in are as follows: with superoxide (reaction 1: k_1_ = 1.9 × 10^10^ M^−1^ s^−1^) [140], with ubiquinol (reaction 2: k_2_ = 4 × 10^3^ M^−1^ s^−1^) [135] and with cytochrome oxidase (reaction 3: k_3_ = 4 × 10^7^ M^−1^ s^−1^) [141]. In aerobic settings, about 85% of the rate of mitochondrial NO use is explained by the fast reaction of NO with O_2_ (diffusion-controlled rate). Strong oxidizing and nitrating species peroxynitrite (ONOO^−^) are formed because of the reaction. Peroxynitrous acid (ONOOH; pKa = 6.8) [140], is produced when ONOO-protonates at physiological pH. After rearranging into an intermediate [HO·····NO2], peroxynitrous acid splits and produces the free radicals HO· and ·NO2 [142]. Ubisemiquinone, which functions as a free radical reaction center capable of producing O_2_^−^ by autooxidation in a propagation process, is created when NO reacts with ubiquinol. Like the interactions of cytochrome oxidase with carbon monoxide (CO), the addition reaction of NO with cytochrome oxidase blocks the primary pathway for the intake of oxygen and the creation of energy. Nitroxyl anion (NO^−^) can be produced in a sluggish first order reaction by the a3-heme transferring one electron to NO. K1, K2 and K3 indicate the rate of NO reaction with O_2_^−^, Cyt a_3_^2+^ and ubiquinol ^(^UQH_2_). In K1 NO reacts with O_2_^−^ at a higher rate than the rate it reacts with Cyt a_3_^2+^, but it reacts with ubiquinol (UQH_2_) at a far lower rate [137], Figure 5.

**Figure 6:**
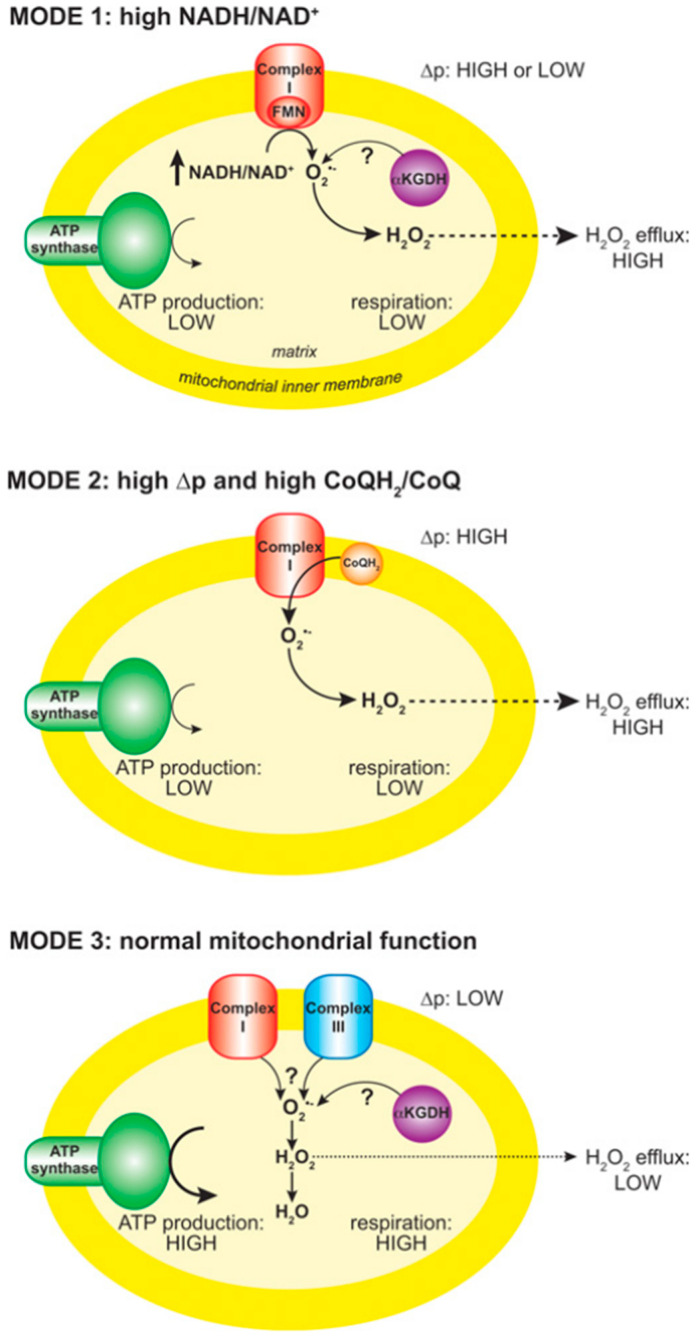
Modes of mitochondrial operation that lead to O_2_^• −^ production. There are three modes of mitochondrial operation that are associated with O_2_^• −^ production. In mode 1, the NADH pool is reduced, for example by a damage to the respiratory chain, loss of cytochrome c during apoptosis or low ATP demand. This leads to a rate of O_2_^• −^ formation at the FMN of complex I that is determined by the extent of FMN reduction which is in turn set by the NADH/NAD^+^ ratio. In mode 2, there is no ATP production and there is a high proton motive force and a reduced CoQ (Ubiquinone) pool which leads to reverse electron transport (RET) through complex I, producing large amounts of O_2_^• −^. In mode 3, mitochondria are actively making ATP and consequently have a lower change in membrane proton motive (MPM) force than in mode 2 and a more oxidized NADH pool than in mode 1. Under these conditions, the flux of O_2_^• −^ within mitochondria is far lower than in modes 1 and 2, and the O_2_^• −^ sources are unclear [143].

The formation of O_2_^• −^ within the mitochondrial matrix is determined by factors such as proton motive force, nicotinamide adenine dinucleotide in reduced and oxidized form (NADH/NAD+) and reduced form of coenzyme Q (ubiquinol)/coenzyme Q in its oxidized state (ubiquinone) (CoQH_2_/CoQ)) ratios, and local O_2_ concentration [143], Figure 6. Mitochondria function in three ways related to O_2_^• −^ production, with isolated mitochondria producing a large amount from complex I when not producing ATP due to high proton motive force and decreased CoQ, which leads to RET. The maximum O_2_^• −^ release in mitochondria is under reverse electron transport (RET), though its location remains unknown. Mitochondria with a high NADH/NAD^+^ ratio showed decreased action at the Flavin mononucleotide (FMN) position of complex I. Active ATP production leads to less O_2_^• −^ generation [143], Figure 6. This implies that O_2_^• −^ production will be favored under in vivo settings that result in RET or an accumulation of NADH.

## 7. Conclusions and Future Research Direction

Rabies is a deadly infectious disease caused by the rabies virus, resulting in almost 100% fatality in mammals, including humans. Despite vaccination efforts, it still causes 59,000 deaths annually, highlighting the need for better therapy and vaccine distribution. The virus uses various strategies to evade immune cell detection, with Pathogenic infections inhibit immune response, while nonpathogenic infections promote virus elimination and cell survival.

Developing countries in Africa and Asia struggle with expensive and ineffective multi-dose rabies treatment due to inadequate medical facilities and limited resources. Affordable and effective vaccines are urgently needed to address the social and economic implications caused by this disease.

The RABV P protein is a multifunctional antagonist which can block the activation of immune-stimulatory and antiviral genes. Besides, it interacts with mitochondrial complex I and contributes to mitochondrial malfunction, making it a potential drug target to maintain innate immunity and mitochondrial functions during RABV infections.

Cellular immunity plays a crucial role in combating rabies virus infection in the central nervous system. While innate immune cells are present, peripheral immune cells like B cells and T cells are needed to effectively control the infection despite the potential for tissue damage [46]. To start the adaptive immune response, naïve T cells need to be activated by APCs [47]. The CNS is an immunologically specialized area with distinct anatomical characteristics, such as the absence of specialized APCs like DCs and secondary lymphoid organs like local lymph nodes, as well as the presence of mechanical barriers like BBB and blood–cerebrospinal fluid barriers that limit the immune system’s access to the CNS. Future research focusing on reducing these barriers and allowing humoral and cell-mediated cells to infiltrate the CNS could potentially prevent neuro-invasion and disease progression.

The RABV p protein and M protein inhibit mitochondrial complex I and cytochrome c oxidase activity, respectively. High concentrations of NO during RABV infection compete with oxygen for cytochrome c oxidase. These interactions lead to increased mitochondrial membrane potential and low cellular ATP production. There is also a buildup of high NADH/NAD and CoQH2/CoQ ratio, which resulted in ascended induction of reactive oxygen species, causing oxidative stress and neurons process degeneration. More research is needed to add an input to the existing knowledge and expand it further to understand the functional and structural changes on the mitochondrial components.

More efforts are needed to manipulate the rabies virus genome for vaccine production and ensure biosafety while maintaining immunogenicity. Live-attenuated viruses have been successful in other diseases but have only been approved for animal use in rabies due to high mortality rates. By using reverse genetic techniques, highly attenuated live rabies virus strains with increased immunogenicity could be developed for human vaccines. This approach could have significant benefits in preventing rabies and other viral diseases. Future research and investment in this area are essential to further manipulate the rabies virus genome and develop highly attenuated strains with increased immunogenicity for effective vaccine development [63].

A study by Xue Feng Niu et al. [64] cloned chemokine genes into the rabies virus genome to understand their role in pathogenesis and protection. Recombinant RABVs expressing CCL5 and CXCL10 induce high and persistent expression of chemokines, leading to disease development and death in mice. However, recombinant RABVs expressing MIP-1a, MDC, and GM-CSF attenuate RABV by promoting transient chemokine expression and enhancing blood–brain barrier permeability. These recombinant RABVs expressing cytokines/chemokines also showed increased adaptive immune responses, potentially preventing rabies days after infection. Further research efforts in this area is worthy enough to build upon the promising outcomes mentioned above.

Rabies and lyssavirus infections are lethal once they enter the central nervous system. F11, a human monoclonal antibody, is being assessed by Mastraccio et al. [65] for therapeutic efficacy against established lyssavirus infections. A single dose of F11 reduces viral load in the brain and reverses clinical signs after infection with a deadly dose of pathogenic lyssavirus. The study also discovered that an adaptive immune response using CD4 T cells is crucial for managing the infection, and F11’s ability to bind FcRc improves treatment effectiveness. This promising preliminary result paves a way for future research.

This review recaps the current understanding of rabies and provides an insight into its subversive strategy to bypass the immune response and pathogenicity mechanisms of several RABV strains. It can advance the ongoing effort to develop effective, safe, and affordable vaccines and drugs that can fully treat rabies.

## Abbreviations

**RABV**: Rabies virus; **CVS**, challenge virus standard; **DUVV**, Duvenhage lyssavirus; **EBLV-1,2**, European bat lyssaviruses type 1 and 2; **ABLV**, Australian bat lyssavirus; **LBV**, Lagos bat lyssavirus; **SHBRV**, silver haired bat-associated rabies virus; **MOKV**, Mokola lyssavirus; **PV**, Pasteur Virus vaccine strain; **SAD-B19**, Street Alabama Dufferin strain; **PM**, Pitman-Moore strain; **CVS-F3**, challenge virus standard, escape mutant; **CVS-N2c**, challenge virus standard; **wt**, wild type; **Cco1**, cytochrome c oxidase subunit 1; **CNS**, central nervous system; **PNS**, peripheral nervous system; **BBB**, blood–brain barrier; **INOS**, inducible nitric oxide synthase (**NOS2**, nitric oxide synthase 2); **eNOS**, endothelial nitric oxide synthase (**NOS3**, nitric oxide synthase 3); **nNOS**, neuronal nitric oxide synthase (**NOS1**, nitric oxide synthase 1); **mNOS**, mitochondrial nitric oxide synthase; **eIF3h**, eukaryotic translation initiation factor 3; **GAS**, γ-activated sequence; **IFNA**, type-1 IFN A; **IKKε**, I-kappa B kinase ε; **TBK1**, TANK-binding kinase 1; **IRF-3**, interferon regulatory factor 3; **IRF-7**, interferon regulatory factor 7; **ISGF3**, IFN-stimulated gene factor 3; **ISGs**, interferon-stimulated genes; **ISRE**, IFN-stimulated response element; **Jak-STAT**, Janus kinase-signal transducer and activator of transcription; **N**, nucleoprotein; **P**, phosphoprotein; **M**, matrix protein; **G**, glycoprotein; **L**, polymerase (large protein); **RNP**, ribonucleoprotein; **mRNA**, messenger RNA; **NBs**, Negri bodies; **PML**, promyelocytic leukaemia; **RdRp**, RNA-dependent RNA polymerase; **RIG-I**, retinoic-acid inducible gene I; **FAK**, focal adhesion kinase; **RING**, really interesting new gene; **RNase**, ribonuclease; **ROS**, reactive oxygen species; **RNS**, reactive nitrogen species; **STAT1/2**, signal transducer and activator of transcription ½; **p75NTR**, low-affinity nerve-growth factor receptor; **NCAM**, neural cell adhesion molecule; **nAChR**, nicotinic acetylcholine receptor; **MAVS**, mitochondrial antiviral signaling protein; **NF-kB**, Nuclear factor kappa B; **PI3K**, phosphoinositide 3-kinase; **PTEN**, phosphatase and tensin homolog; **PTPN4**, tyrosine-protein phosphatase non-receptor type 4; **MAST2**, microtubule-associated serine/threonine protein kinase 2; **MTOR**, mechanistic target of rapamycin; **GM-CSF**, granulocyte macrophage colony stimulating factor; **PRRs**, pattern-recognition receptors; **PAMPs**, pathogen-associated molecular patterns; **DAMPs**, damage-associated molecular patterns; **Ab**, antibody; **MHC**, major histocompatibility complex; **pSTAT1**, phosphorylated(activated) Signal transducer and activator of transcription 1; **NES**, nuclear export signal; **NLS**, nuclear localization sequence; **CTD’s**, The C-terminal domain; **HPA**, hypothalamic pituitary adrenal; **ERA**, Evelyn-Rokitnicki-Abelseth; **DRV-4**, dog rabies virus; **Thai-DRV**, Thailand genotype 1 dog lyssavirus; **CosRV**, coyote street rabies virus; **HEP-flurry**, high-egg-passage Flury strain; **TJ proteins**, tight junction proteins; **CCL5**, C-C motif chemokine ligand 5 (RANTES); **CXCL10**, C-X-C motif chemokine 10 (IP-10); **IP-10**, Interferon-gamma inducible Protein 10kDa; **MCP-1**, Monocyte chemoattractant protein-1; **CXCR3**, C-X-C motif chemokine receptor; **CD4**, cluster of differentiation 4; **CD8**, cluster of differentiation 8; **VNAs**, virus-neutralizing antibodies; **PEP**, post exposure prophylaxis; **MIP-1a**, Macrophage inflammatory protein-1α; **MDC**, Macrophage-derived chemokine; **TNFR1**, tumor necrosis factor receptor type 1; **TRAIL**, tumor necrosis factor-related apoptosis-inducing ligand; **cFLIP**, cellular FLICE-like inhibitory protein; **Bcl-2**, B-cell lymphoma 2; **Bcl-xL**, B-cell lymphoma-extra-large; **Cyt c**, cytochrome c; **SMAC**, Second mitochondria-derived activator of caspase; **PD-1**, Programmed death-ligand 1; **B7-H1**, B7 homolog 1; **HLA-G**, human leukocyte antigen G; **RET**, reverse electron transport; **FMN**, Flavin mononucleotide (FMN), or riboflavin-5′-phosphate; **siRNAs**, short interfering RNAs; **ATP**, Adenosine triphosphate; **NADH**, nicotinamide adenine dinucleotide (NAD)+ hydrogen (H); **CoQH2 (Ubiquinol/UQH2)**, reduced coenzyme Q; **Cu-Zn-SOD**, copper-zinc-superoxide dismutase; **CoQ (ubiquinone/UQ)**, oxidized coenzyme Q; **Mn-SOD**, manganese-superoxide dismutase; **mtNOS**, mitochondrial nitric oxide synthase; **APCs**, antigen-presenting cells; **FcRc**, Fc receptor; **HIAP**, Human Inhibitor of Apoptosis Protein; **NADPH**, nicotinamide adenine dinucleotide phosphate; **NK** cells, natural killer cells; **AG**, Amino-guanidine; **BECN1**, Beclin-1; **CASP2**, Caspase 2; **AMPK**, Adenosine monophosphate-activated protein kinase; **MAPK**, Mitogen-activated protein kinase; **AKT**, Protein kinase B; **SK cells**, human neuroblastoma cell line; **Bax**, Bcl-2-associated X protein; **Bad**, BCL2 associated agonist of cell death; **STAT3**, signal transducer and activator of transcription 3; **Apaf-1**, apoptotic protease activating factor-1; **GPR17**, G Protein-Coupled Receptor 17; **BAK**, Bcl-2 antagonist killer 1; **LC3-I**, Microtubule-associated protein light chain 3-I; **AIF**, Apoptosis-inducing factor; **BH3**, Bcl-2 homology domain 3; **YFP**, yellow fluorescent protein; **PARP**, poly-ADP ribose polymerase; **DRG**, dorsal root ganglion; **PC12**, pheochromocytoma (cancer) cell line; **MNA**, Mouse Neuroblastoma cell line; **CcO**, Cytochrome c oxidase; **Slc25a4**, Solute Carrier Family 25 Member 4; **NAD^+^**, Nicotinamide adenine dinucleotide; **ETS**, electron transport system; **MMP**, mitochondrial membrane potential; **SOD_2_**, superoxide dismutase; **H_2_O_2_**_,_ hydrogen peroxide; **O_2_·^−^**, superoxide anion; **ONOO^−^**, Peroxynitrite; **DHR123**, 5.7.2 Dihydrorhodamine 123; **SNAP**, Soluble N-ethylmaleimide-Sensitive Factor Attachment Proteins; **DCFH-DA**, 2′−7′-dichlorodihydrofluorescein diacetate; **EPC-K1**, ascorbyl tocopheryl phosphate; **nM**, nano mole; **μM**, micro mole; **pKa**, acid dissociation constant; **CO**, carbon monoxide; **UQH_2_(CoQH2)**, ubiquinol/reduced form of coenzyme Q.; **HO**, hydroxyl radical; **HO^−^**, hydroxyl ion; **NO**, nitric oxide radical, **NO^−^**, nitroxyl anion; **NO_2_**, nitrogen dioxide; **NO_2_**, nitric dioxide radical; **NO_2_^−^**, nitrous acid(nitrite).
